# Enhancing Disease Detection in Electronic Medical Records: Integrating Human Expertise and Large Language Models with Application to Diabetes, Hypertension, and Acute Myocardial Infarction

**DOI:** 10.23889/ijpds.v9i5.2633

**Published:** 2024-09-18

**Authors:** Jie Pan, Seungwon Lee, Cheligeer Cheligeer, Elliot Martin, Kiarash Riazi, Hude Quan, Na Li

**Affiliations:** 1 Centre for Health Informatics, Cumming School of Medicine, University of Calgary; 2 Department of Community Health Sciences, Cumming School of Medicine, University of Calgary; 3 Libin Cardiovascular Institute, University of Calgary; 4 Provincial Research Data Services, Alberta Health Services

## Objective

Electronic medical records (EMR) are widely available to complement administrative data-based disease surveillance and healthcare performance evaluation. Defining conditions from EMR is labour-intensive, requiring advanced medical informatics knowledge, and is challenging without effective data extraction tools. This study developed a high-throughput pipeline to detect diseases in EMRs.

## Methods

We developed a pipeline that leverages a generative large language model (LLM) to analyze, understand, and interpret EMR notes by following clinical experts’ designed prompts. The pipeline was applied to detect diabetes, hypertension, and acute myocardial infarction (AMI) from the EMRs for a cardiac patient cohort in Calgary, Canada. The performance was compared against clinician-validated diagnoses as the reference standard.

## Results

The cohort consisted of 3,413 patients with 551,095 clinical notes. The prevalence was 27.8%, 66.3%, and 54.3% for diabetes, hypertension, and AMI, respectively. The performance for detecting conditions varied: diabetes had 90.5% sensitivity, 83% specificity, and 67% positive predictive value (PPV); hypertension had 94.2% sensitivity, 30.2% specificity, and 73.8% PPV; and AMI had 86.4% sensitivity, 61% specificity, and 75.3% PPV. The monthly prevalence trends between the detected cases and reference standard showed similar patterns.

## Conclusion

The proposed pipeline demonstrated reasonable accuracy and high efficiency in disease detection without manually curated labels, indicating the potential for automated real-time disease surveillance using EMRs.

## Implication

Variations of documentation practices in clinical note can impact the detection performance of different diseases. Hence, an automated pipeline integrating LLMs with expert knowledge may improve detection accuracy with reduced labour costs while indicating documentation quality.

